# Fe Porphyrin-Based SOD Mimic and Redox-Active Compound, (OH)FeTnHex-2-PyP^4+^, in a Rodent Ischemic Stroke (MCAO) Model: Efficacy and Pharmacokinetics as Compared to Its Mn Analogue, (H_2_O)MnTnHex-2-PyP^5+^

**DOI:** 10.3390/antiox9060467

**Published:** 2020-06-01

**Authors:** Litao Li, Artak Tovmasyan, Huaxin Sheng, Bin Xu, Romulo S. Sampaio, Julio S. Reboucas, David S. Warner, Ines Batinic-Haberle, Ivan Spasojevic

**Affiliations:** 1 Multidisciplinary Neuroprotection Laboratories, Departments of Anesthesiology, Biomedical Engineering, Neurobiology, and Neurosurgery, Duke University School of Medicine, Durham, NC 27710, USA; dingding51800@163.com (L.L.); huaxin.sheng@duke.edu (H.S.); xubin@mail.cmu.edu.cn (B.X.); david.warner@duke.edu (D.S.W.); 2 Department of Radiation Oncology, Duke University School of Medicine, Durham, NC 27710, USA; Artak.Tovmasyan@BarrowNeuro.org (A.T.); ibatinic@duke.edu (I.B.-H.); 3 Departamento de Química, CCEN, Universidade Federal da Paraíba, João Pessoa, PB 58051-900, Brazil; romulo.smp@gmail.com (R.S.S.); jsreboucas2@gmail.com (J.S.R.); 4 Department of Medicine, Duke University School of Medicine, Durham, NC 27710, USA; 5 PK/PD Core Laboratory, Duke Cancer Institute, Duke University School of Medicine, Durham, NC 27710, USA

**Keywords:** MnTnHex-2-PyP^5+^, FeTnHex-2-PyP^5+^, SOD mimics, pharmacokinetics, rodent middle cerebral artery occlusion, MCAO

## Abstract

Mn(III) *meso*-tetrakis(*N*-n-hexylpyridinium-2-yl)porphyrin, (H_2_O)MnTnHex-2-PyP^5+^ (MnHex) carrying long hexyl chains, is a lipophilic mimic of superoxide dismutase (SOD) and a redox-active drug candidate. MnHex crosses the blood–brain barrier, and improved neurologic outcome and decreased infarct size and inflammation in a rat middle cerebral artery occlusion (MCAO) ischemic stroke model. Yet, the dose and the therapeutic efficacy of Mn porphyrin were limited by an adverse effect of arterial hypotension. An equally lipophilic Fe analog, (OH)FeTnHex-2-PyP^4+^ (FeHex), is as redox-active and potent SOD mimic in vitro. With different coordination geometry of the metal site, FeHex has one hydroxo (OH) ligand (instead of water) bound to the Fe center in the axial position. It has ~2 orders of magnitude higher efficacy than MnHex in an SOD-deficient *E. coli* model of oxidative stress. In vivo, it does not cause arterial hypotension and is less toxic to mice. We thus evaluated FeHex *versus* MnHex in a rodent MCAO model. We first performed short- and long-term pharmacokinetics (PK) of both porphyrins in the plasma, brain, and liver of rats and mice. Given that damage to the brain during stroke occurs very rapidly, fast delivery of a sufficient dose of drug is important. Therefore, we aimed to demonstrate if, and how fast after reperfusion, Fe porphyrin reaches the brain relative to the Mn analog. A markedly different plasma half-life was found with FeHex (~23 h) than with MnHex (~1.4 h), which resulted in a more than 2-fold higher plasma exposure (AUC) in a 7-day twice-daily treatment of rats. The increased plasma half-life is explained by the much lower liver retention of FeHex than typically found in Mn analogs. In the brain, a 3-day mouse PK study showed similar levels of MnHex and FeHex. The same result was obtained in a 7-day rat PK study, despite the higher plasma exposure of FeHex. Importantly, in a short-term PK study with treatment starting 2 h post MCAO, both Fe- and Mn- analogs distributed at a higher level to the injured brain hemisphere, with a more pronounced effect observed with FeHex. While a 3-day mouse MCAO study suggested the efficacy of Fe porphyrin, in a 7-day rat MCAO study, Mn-, but not Fe porphyrin, was efficacious. The observed lack of FeHex efficacy was discussed in terms of significant differences in the chemistry of Fe vs. the Mn center of metalloporphyrin; relative to MnHex, FeHex has the propensity for axial coordination, which in vivo would preclude the reactivity of the Fe center towards small reactive species.

## 1. Introduction

**Figure 1:**
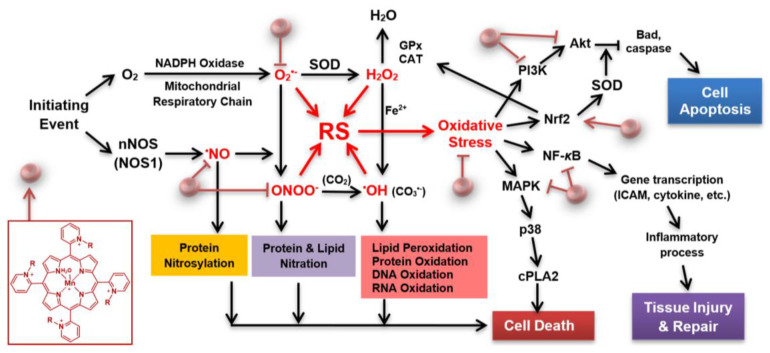
Superoxide dismutase (SOD) porphyrin-based mimic and cell/death survival pathways. Porphyrin-based SOD mimic blocks reactive species (RS) (directly or indirectly) produced upon blockade of the cerebral blood flow, which would have otherwise modified redox-sensitive cellular pathways leading to the excessive inflammation and in turn cellular death [1,2]. An SOD mimic would activate Nrf2 (nuclear factor E2-related factor 2), while suppress activities of NF-кB (nuclear factor кB), and MAPKs (mitogen-activated protein kinases), such as p38 MAPK (p-38 mitogen-activated protein kinase), JNK (c-Jun N-terminal kinases), and ERK (extracellular signal-regulated kinase) [1,2]. Activation of Nrf2 by porphyrin-based SOD mimic would result in upregulation of endogenous antioxidative defenses, such as MnSOD, catalase and GPx, which would result in suppression of oxidative stress. Other abbreviations: R in porphyrin structure may be ethyl [in (H_2_O)MnTE-2-PyP^5+^)], hexyl [in (H_2_O)MnTnHex-2-PyP^5+^] or butoxyethyl [in (H_2_O)MnTnBuOE-2-PyP^5+^)], CAT (catalase), cPLA2 (phospholipase A2), PI3K (phosphoinositide 3-kinases), ICAM (intercellular adhesion molecule), GPx (glutathione peroxidase), nNOS (neuronal nitric oxide synthase), BAD (pro-apoptotic protein from Bcl-2 family of proteins). Modified from [3].

Currently, there are no effective drugs for stroke other than thrombolytics. In the early phase of stroke, mitochondria contribute to the production of reactive species. Thus, therapeutics able to accumulate in mitochondria, and rapidly enough, are preferable. The superoxide dismutase family of enzymes (mitochondrial MnSOD, cytosolic and mitochondrial intermembrane space Cu, ZnSOD isoform) plays a key role in ischemic stroke, maintaining the physiological redox status of the cell. The SOD enzymes, via metal centers, catalyze dismutation of superoxide (O_2_^•−^) into oxygen and hydrogen peroxide (H_2_O_2_). In stroke injury, these enzymes could be oxidatively damaged, thus inactivated, or their levels downregulated. The rise in reactive species, such as O_2_^•−^, H_2_O_2_, nitric oxide (^•^NO), and peroxynitrite (ONOO^−^), leads to oxidative stress, which in turn activates transcription factors, such as NF-кB, Nrf2, pro-apoptotic protein BAD, caspases, and other signaling molecules, including mitogen-activated protein kinases, MAPKs (such as AKT, PI3K, p38 MAPK). This course of events results in inflammation, which if excessive leads to tissue damage and eventually to cell apoptosis (Figure 1). 

Our focus has been on the study of redox-active metal complexes as therapeutics, which can penetrate cellular and mitochondrial membranes and cross the blood–brain barrier (BBB). We developed cationic complexes of Mn(III) with *N*-alkyl or *N*-alkoxyalkyl-substituted pyridylporphyrins; a cyclic porphyrin ligand assures the extreme stability of the metal complex and thus the integrity of the metal site where redox reactions of interest occur [4,5,6,7]. The potency of those compounds approaches that of SOD enzymes. Like SOD enzymes, Mn porphyrins suppress oxidative stress and excessive inflammation via direct reactions with reactive species (e.g., O_2_^•−^, ^•^NO, and OONO^−^), but also indirectly via redox modulation of transcription factors, most so NF-кB, Nrf2 and MAPKs [1,2,6,8,9]. Redox modulation occurs via Mn porphyrin-driven catalysis of oxidation/*S*-glutathionylation of cysteines of signaling proteins in the presence of H_2_O_2_ and glutathione [1,6].

**Figure 2:**
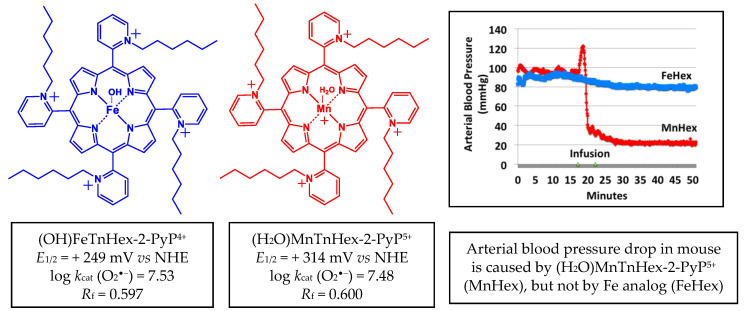
Structures of aqua Mn(III) *meso*-tetrakis(*N*-n-hexylpyridinium-2yl)porphyrin, (H_2_O)MnTnHex-2-PyP^5+^ and monohydroxo Fe(III) *meso*-tetrakis(*N*-n-hexylpyridinium-2yl)porphyrin, (OH)FeTnHex-2-PyP^4+^. Redox properties that determine the ability of these compounds to exhibit therapeutic effects are listed: metal centered reduction potential for M^III^/M^II^ redox couple, *E*_1/2_ in mV vs. NHE (*M* = metal) and log value of the rate constant for the metalloporphyrin-driven catalysis of O_2_^•^^−^dismutation, log *k*_cat_(O_2_^•^^−^). Lipophilicities are also listed, expressed in terms of the thin-layer chromatographic *R*_f_, which shows the path of the compound vs. the path of the solvent on silica plates in acetonitrile/KNO3(sat)/water = 8:1:1. Data are taken from [13]. At an identical intraperitoneal (IP) dosing of 10 mg/kg, MnHex does cause a blood pressure drop in mice, while FeHex does not (modified from [13]).

The early generation redox-active and lipophilic cationic compound (H_2_O)MnTnHex-2-PyP^5+^ (MnHex) (Figure 2) crosses the BBB [10] and accumulates in mitochondria 3.6-fold more than in cytosol [11]. It has been proven to be efficacious in numerous therapeutic studies in vitro and in vivo, which are summarized in [5,12]. It also improved the neurologic outcome and decreased infarct size and inflammation in a rat middle cerebral artery occlusion (MCAO) stroke model and subarachnoid hemorrhage [9]. Yet, the therapeutic efficacy of Mn porphyrin in stroke is limited to only 0.225 mg/kg per injection due to an adverse effect on arterial hypotension (Figure 2).

Along with Mn-, we also developed and characterized Fe(III) *N*-alkypyridylporphyrins [4,13]. They are equally potent and equally lipophilic SOD mimics and redox-active drugs as their Mn analogs [4,13], but they have a different coordination geometry of the metal site; while the Mn center has weakly coordinated water, the Fe center has a strongly coordinated hydroxo ligand [4]. We fully characterized both Fe and M porphyrins on their chemical and physical properties [4,13]. While MnHex was tested on its efficacy in different cellular and animal models of normal tissue injury and cancer [5,12], the therapeutic efficacy of (OH)FeTnHex-2-PyP^4+^ (FeHex) was only assessed in an *E. coli* screening model of oxidative stress [13]. Due to the different molecular structure, FeHex, with an axially bound hydroxo ligand, does not cause arterial hypotension in experimental animals. It is further less toxic to mice, and showed a ~2 orders of magnitude higher efficacy in an SOD-deficient *E. coli* model of oxidative stress [13]. While mice died from a 5 mg/kg IP injection of Mn analog, the same dose of Fe porphyrin showed no toxicity [13]. Given the high redox potency and high lipophilicity of FeHex, the lack of arterial hypotension, and lower toxicity, which limits the dosing of Mn porphyrin, we hypothesized that the use of Fe porphyrin would allow us to markedly improve the therapeutic efficacy in ischemic stroke. We first assessed the comprehensive pharmacokinetics of FeHex vs. MnHex in rat and mouse, followed by an evaluation of their therapeutic potential in rat and mouse ischemic MCAO stroke model where the right hemisphere was occluded.

## 2. Experimental

Our approach was to first assess the pharmacokinetics (PK) of MnP and FeP under an identical regimen in the plasma, brain, and liver of mice and rats. The development of the LCMS/MS methodology for FeHex and MnHex preceded pharmacokinetic studies. We used the stroke models in both mice and rats, which we developed and reviewed in [14].

## 3. Materials and Methods

FeHex and MnHex were synthesized as described [13]. Other chemicals used were acetonitrile (MeCN) by Fisher Scientific, methanol (anhydrous, absolute) by Mallinckrodt, liquid nitrogen and glacial acetic acid by EM Science, heptafluorobutyric acid (HFBA) by Aldrich, and phosphate-buffered saline (50 mM sodium phosphate, 0.9% NaCl, pH 7.4) by Gibco.

### 3.1. Animals

Duke University Medical Center Animal Facility has a continuously accredited program from AAALAC International. All experiments using animals were performed according to the approved protocol for humane care and use of animals. We performed studies on mice and rats, following the requirement of the preclinical stroke research where the efficacy should be tested in different species. The 10-week-old C57BL/6J female mice weighing 17–25 g, purchased from Jackson Laboratory, and 250–275 g Wistar rats, purchased from Envigo RMS, Inc., were used throughout the study. No change in animal weights larger than 10% was recorded in all studies.

### 3.2. LC-MS/MS Analysis of MnHex and FeHex

**Figure 3:**
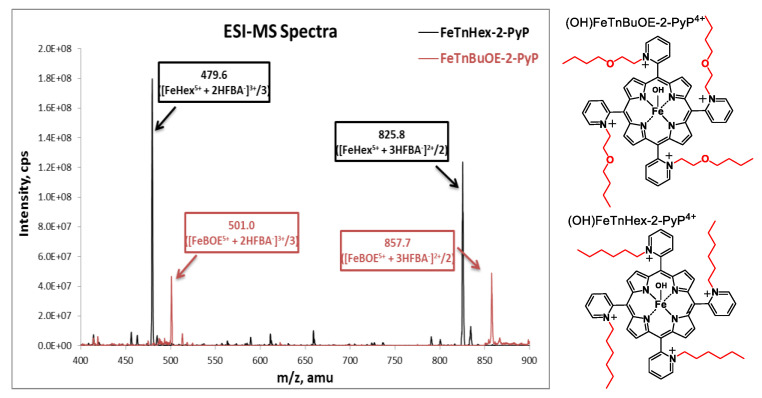
Electrospray-ionization mass spectrometry (ESI-MS) spectra of (OH)FeTnHex-PyP^4+^ (FeHex) and its internal standard (OH)FeTnBuOE-2-PyP^4+^. The main m/z ions are identified as [FeP^5+^ + 2HFBA^−^]^3+^ and [FeP^5+^ + 3HFBA^−^]^2+^ for both FeHex and internal standard, (OH)FeTnBuOE-2-PyP^4+^ (FeBOE), and were further used in the LC-MS/MS analyses of samples. When m/z ions are listed, FeP stands for Fe porphyrin and refers to both FeHex and the internal standard. The structures of (OH)FeTnBuOE-2-PyP^4+^ and (OH)FeTnHex-PyP^4+^ are also shown. The axial hydroxo ligands, shown in structures, are lost/defragmented during electrospray ionization/declustering process, resulting in the ion mass indicated.

Samples were prepared as described in [10] with modifications to accommodate the small tissue sizes and enhance the detection sensitivity. Quantitative liquid chromatography electrospray-ionization tandem mass spectrometry (LCMS/MS) analysis was performed on a Shimadzu 20A series HPLC (LC)—Applied Biosystems/MDS Sciex API 3200 QTrap or API 4000 QTrap tandem-mass spectrometer (MS/MS) as described earlier [15]. The use of heptafluorobutyric acid (HFBA) mobile phase modifier as an ion-pairing agent increases the overall lipophilicity and volatility, which increases the retention and ionization efficiency of the analytes, affording an abundance of HFBA-associated ions. (OH)FeTnBuOE-2-PyP^4+^ and (H_2_O)MnTnBuOE-2-PyP^5+^ were used as internal standards for FeHex and MnHex; introduction of an oxygen atom into each of the four *N*-n-hexylpyridyl chains of (H_2_O)MnTnHex-2-PyP^5+^ or (OH)FeTnHex-2-PyP^4+^ (shown in Figure 2) resulted in the formation of analogs with four *N*-n-butoxyethylpyridyl chains, (H_2_O)MnTnBuOE-2-PyP^5+^ and (OH)FeTnBuOE-2-PyP^4+^ [6,13,16]. Since oxygen atoms are buried within the porphyrin cavity and thus not easily solvated, these compounds maintain similar redox properties and lipophilicity as their parent hexyl analogs, which makes them co-elute from the HPLC column and thus can be used as internal standards [6,13,16]. The structures of Fe analogs are shown in Figure 3. (OH)FeTnBuOE-2-PyP^4+^ was prepared as described for other Fe(III) *N*-alkylpyridylporphyrins and analogous (H_2_O)MnTnBuOE-2-PyP^5+^ [13,16]. The solvents employed were: A = 95:5 H_2_O:MeCN (0.1% HFBA); B = MeCN (0.1% HFBA). Both MnHex and FeHex, like all *ortho* Mn(III) *N*-substituted pyridylporphyrins, exist as a mixture of four atropoisomers [17]. With the chosen solvent system, all four atropoisomers collapsed into a single peak, enhancing the sensitivity of the method to as low as 0.5 nM (nanomoles per L of tissue homogenate). Analyses were performed using a Phenomenex Luna C18 guard cartridge (ID × L, 2 × 4 mm) only. Specific ions, m/z [MP^5+^ + 3HFBA^−^]^2+^ and m/z [MP^5+^ + 2HFBA^−^]^3+^, were followed (M = Mn or Fe, P = porphyrin) as presented for FeHex and its standard (OH)FeTnBuOE-2-PyP^4+^ (FeBOE) in Figure 3. Their structures are also shown. Calibration samples in the 1–300 nM or 0.1–30 μM range (depending on the expected levels of MP) were prepared by adding known amounts of serially diluted pure standards into the plasma or homogenates of related tissues. The response was calculated as the ratio between the standard peak area and the internal standard peak area.

### 3.3. Pharmacokinetic (PK) Studies

Short- (2-hourand long-term (3-day and 7-day) PK studies were performed in rats and mice with and without MCAO with the ultimate goal of determining which levels of FeHex and MnHex in the brain could protect it from stroke ischemia/reperfusion injury. In addition to the brain, plasma PK was followed and levels in the liver determined. Liquid chromatography electrospray-ionization tandem mass spectrometry, LCMS/MS, was used as described above. Two animal species were used for both the PK and efficacy studies to comply with the requirements for the use of different species in preclinical stroke research.

#### 3.3.1. The 3-Day PK Study in Healthy Mice

The study was designed to follow the dosing regime in a 3-day mouse MCAO efficacy study. Mice were given FeHex and MnHex every 12 h for 3 days via 2 routes: Intravenously (IV) at 0.5 mg/kg and subcutaneously (SC) at 0.5 mg/kg (for a total of 3 mg/kg). Three mice were used per compound in each study. Mice were sacrificed 6 h after the last injection at day 3 and blood and tissues (liver and brain) collected. Blood was centrifuged at 2500 rpm for 10 min and plasma was harvested. Plasma and tissues were analyzed on porphyrin content by LCMS/MS.

#### 3.3.2. The 7-Day PK Study in Healthy Rats

We conducted a 7-day rat PK study under conditions identical to those that afforded the therapeutic efficacy of MnTnHex-2-PyP^5+^ in an earlier study [9]. FeHex and MnHex were given to rats SC for 7 days every 12 h at 0.225 mg/kg. FeHex plasma PK within the first 12 h was done at 10 min, 30 min, and 1 h, 2 h, 4 h, and 12 h after the 1st injection. Afterwards, blood/plasma samples were collected before injection and at 2 h after 24 h-injections (every second injection). Liver and brains were taken for analysis 12 h after the last injection. Three rats were used per compound.

#### 3.3.3. The 2-h PK Study in Rats that Underwent MCAO

Another possible cause for the failure of therapeutics in stroke may be their slow accumulation in the brain, i.e., not being there in time to prevent the burst of reactive species and rapid activation of transcription factors, which perpetuate oxidative stress [9,18,19]. We therefore conducted short-term 2-h PK to evaluate how fast post-MCAO our drug gets into the brain. Three rats per compound were subjected to 90 min of transient right MCAO as reported [9]. The first dose of 0.225 mg/kg was given IV via the tail artery at 5 min after ischemia/reperfusion. The second dose of 0.225 mg/kg was given SC at 30 min after reperfusion. Blood was collected at 2 h and plasma harvested. Rats were perfused with heparin saline (2000 IU/500 mL) until no reddish fluid came out from the right atrium. Then, the liver, and left and right brain were collected and placed on dry ice and stored at −20 °C. Plasma, liver, and brain were analyzed by LCMS/MS.

### 3.4. Efficacy MCAO Studies

#### 3.4.1. The 3-Day Mouse MCAO Study

We performed a mouse MCAO study to define a dose of FeHex for a larger rat MCAO study. The 81 C57Bl/6J mice (8–10 weeks old) were subjected to 90 min of MCAO right hemisphere occlusion [20]. Mice were fasted overnight before injury. They were under isoflurane anesthesia during ischemia, and the pericranial temperature was automatically controlled at 37 °C using a surface heating and cooling system. Ischemia was made through a small window on the left temporal bone, and the main trunk of the middle cerebral artery was transiently tied with a fine silk suture for 90 min. Muscle and skin layers were closed after ischemia/reperfusion. Mice were allowed to awake and survive for 3 days. Mice received FeHex twice per day for 3 days IV, via tail vein injections beginning at 90 min after ischemia/reperfusion. Mice were randomly assigned into one of 4 groups: (1) Vehicle (no FeHex); (2) 0.05 mg/kg FeHex; (3) 0.225 mg/kg FeHex; and (4) 1 mg/kg FeHex. Mice were sacrificed under deep anesthesia at 3 days post-ischemia/reperfusion. The brain was quickly frozen in −20 °C 2-methybutane and stored in the freezer for histological analysis. Liver and brain were taken for LCMS/MS analysis of the levels of FeHex. Body weight, rotarod performance, and Corner test were assessed before and 3 days after injury. Neurological deficit (neuroscore) and infarct volume were evaluated at 3 days after injury.

The rotarod test was performed on the day before surgery and 3 days after ischemia/reperfusion. Three trials were performed with a 15-min intra-trial rest. Mice were placed on the rod and then the machine was started. The speed was accelerated from 4 turns/min to 40 turns per min. The latency to fall from the rod was automatically recorded. The data are presented in % and are calculated as [(reading after injury)/(reading before surgery-baseline reading)] × 100. Neurological deficit was assessed based on the neuroscore system we previously used that includes the general status and motor and sensory functions [9]. For the corner test, two clean boards were placed in “V” fashion at an angle of 30° relative to the walking axes and mice were left to walk in towards the apex of the “V”. Mice usually make left turns more frequently when injury is severe enough. The left turn was calculated from the number of left (L) turn/total turns (L + Right turn). Normal animals would have as many left as right turns (50% = normal). When all turns are to the left, the value at y-axis is 100%, the most severe score. Body weight loss is expressed as a difference in body weight at 3 days post-stroke minus body weight on the surgery day.

#### 3.4.2. The 7-Day Rat MCAO Study

The 7-day rat MCAO study was done as described in [9]. The 250–275 g Wistar rats were used as another species is a requirement for preclinical stroke research. The duration of the right MCAO was 90 min. Rats were randomly assigned into one of three groups (*n* = 15–16) and were injected with either saline (vehicle) or drug twice per day for 7 days: (1) Vehicle (saline); (2) FeHex (0.225 mg/kg); and (3) MnHex (0.225 mg/kg). The first dose was given IV slowly via the tail artery at 5 min after ischemia/reperfusion. The following doses were given SC. At day 7 post-injury, a person blinded to the treatment groups assessed the neurological deficit and then brains were harvested for infarct volume measurements.

## 4. Results

### 4.1. Pharmacokinetic Studies

#### 4.1.1. The 3-Day Mouse PK Study

**Figure 4:**
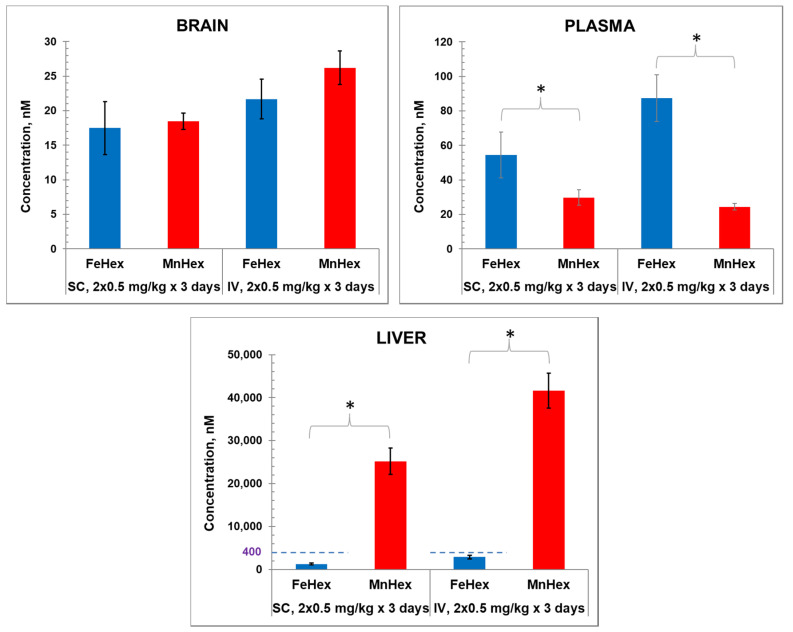
The 3-day mouse PK study. MnHex and FeHex were given twice daily to healthy mice via two administration routes: SC at 0.5 mg/kg and IV at 0.5 mg/kg. Three mice were analyzed per data point and per compound. Mice were sacrificed 6 h after last injection, at which point plasma and tissues were analyzed. Error bars depict single standard deviation. Brain SC, FeHex vs. MnHex, *p* = 0.728; Brain IV FeHex vs. MnHex, *p* = 0.275; Plasma SC FeHex vs. MnHex, *p* = 0.042; Plasma IV, FeHex vs. MnHex, *p* = 0.019; Liver SC, FeHex (126 nm) vs. MnHex, *p* = 0.005; Liver IV, FeHex (290 nm) vs. MnHex, *p* = 0.003. Please note different scale (in blue) for FeHex bars in “Liver” plot. The “star” (*) symbol denotes statistically significant difference (*p* < 0.05).

Porphyrins were administered twice daily via SC and IV injections. Data shown in Figure 4 demonstrate similar levels of Fe Hex and MnHex in the brain when given SC and slightly higher levels when given IV. Higher levels of FeHex than of MnHex were found in plasma, and much lower levels of FeHex relative to MnHex were found in the liver via both SC and IV injections.

#### 4.1.2. The 7-Day Rat PK Study

We conducted the 7-day PK study of FeHex vs. MnHex under conditions similar to those reported [9], when the therapeutic efficacy of MnHex was established. FeHex and MnHex were given SC twice daily (BID) every 12 h to uninjured rats. After the first dose (time zero), plasma levels were measured at several time points for 12 h to assess a single-dose PK profile. Measurements were continued before and 2 h after each subsequent injection.

**Figure 5:**
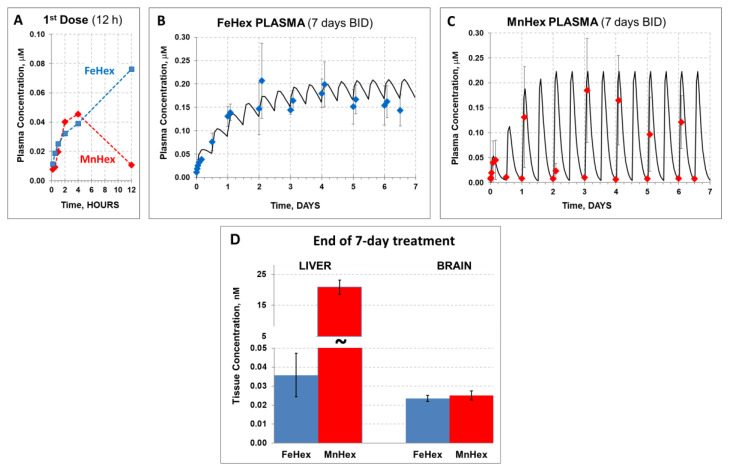
The 7-day rat PK study in healthy rats. FeHex and MnHex were given to rats SC for 7 days every 12 h at 0.225 mg/kg. (**A**) FeHex single plasma PK within the first 12 h was done at 10 min, 30 min, and 1 h 2 h, 4 h, and 12 h after the 1st injection. (**B**,**C**) Plasma levels were followed before and 2 h after each subsequent injection; solid line represents the predicted profile after first-order absorption/first-order elimination simulation by WinNonlin software. (**D**) Liver and brains were taken for analysis 12 h after the last injection. Error bars depict single standard deviation. Liver, FeHex vs. MnHex, *p* = 0.004. Brain, FeHex vs. MnHex, *p* = 0.548.

After the single (first) SC injection, we obtained a prototypical PK profile observed in the case of all Mn(III) *ortho N*-alkypyridylporphyrins [15,21]): Most of the MnHex was eliminated from plasma within 12 h (Figure 5A). In contrast, although similarly absorbed after SC injection, the FeHex 12-h “trough” level was the highest measured, suggesting a much slower elimination from the plasma than in the case of MnHex. This behavior resulted in a markedly different plasma profile in the 7-day 0.225 mg/kg BID treatment (Figure 5B,C). Simulation using first-order absorption, and first-order elimination (a simplified version of the observed multi-compartment tissue distribution process responsible for plasma decay within 12 h) was performed by WinNonlin software to obtain a reasonably close estimate of the area under the concentration/time profile, AUC, and maximal concentration (at a steady state—plateau), C_max_, for discussion purposes. Namely, although C_max_ at a steady state of FeHex and MnHex was very similar (0.21 and 0.22 µM, respectively), the plasma exposure parameters, such as the average concentration (C_aver)_, and AUC, were markedly higher for FeHex (C_aver_ = 0.16 µM, AUC = 26.9 µM∙h) than for MnHex (0.076 µM, AUC = 11.5 µM∙h). In the simulation process, this large difference was obtained by merely changing the presumed elimination rate for FeHex and MnHex from 0.03 to 0.5 s^−1^ (corresponding half-life from 23 to 1.4 h), respectively. Our understanding is that the “elimination” process is actually a fast distribution/retention into/by the liver, kidney, and spleen, a multi-compartmental process responsible for early decay of the plasma concentration, observed for all Mn porphyrin analogs investigated [15,21]. Indeed, end-of-treatment 7-day liver tissue levels of MnHex were very high (21 μM) in contrast with the much lower FeHex levels found (0.04 μM) in support of the lack of a fast tissue distribution process due to liver and other tissue retention (Figure 5D). Notably, both FeHex and MnHex compounds were measurable in the brain (our target) tissue, with very much the same concentration measured at the end of the 7-day treatment, 0.024 and 0.025 μM, respectively, which is very similar to the levels in a 3-day PK study. The brain levels of MnHex of 25 nM were reproduced from an earlier 7-day MCAO study (~30 nM), where it also significantly reduced the infarct size [9].

#### 4.1.3. The 2-h Rat PK Study in an MCAO Model

**Figure 6:**
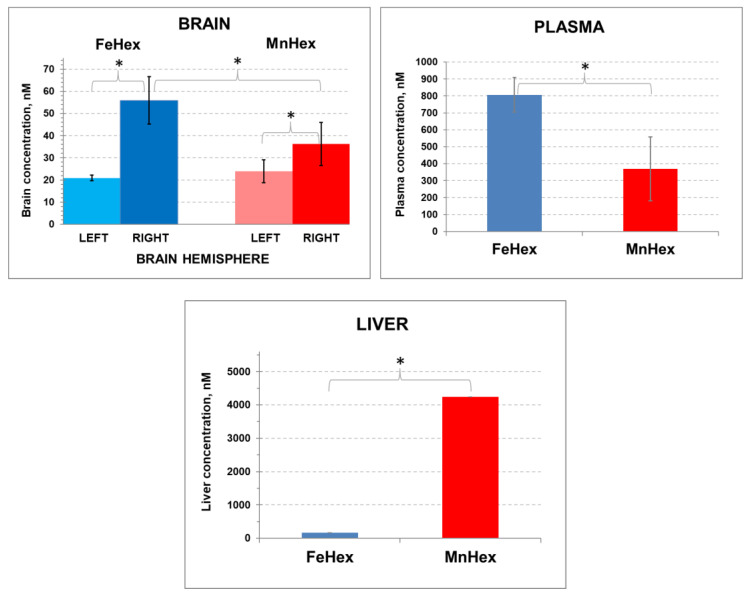
The 2-h rat PK study. Rats underwent 90-min MCAO. The first dose of 0.225 mg/kg was given IV via the tail artery 5 min after reperfusion. The second 0.225 mg/kg dose was given SC at 30 min after reperfusion. The right hemisphere was occluded and underwent ischemia/reperfusion. Blood was collected at 2 h after reperfusion. Rats were perfused for tissue collection. Error bars depict single standard deviation. BRAIN: FeHex, left vs. right brain hemisphere, *p* = 0.024. MnHex, left vs. right hemisphere, *p* = 0.051. MnHex left hemisphere vs. FeHex left hemisphere, *p* = 0.408. MnHex right hemisphere vs. FeHex right hemisphere, *p* = 0.032. PLASMA: FeHex vs. MnHex, *p* = 0.024. LIVER: FeHex vs. MnHex, *p* = 0.016. The “star” (*) symbol denotes statistically significant difference (*p* < 0.05).

In order to assess the kinetics of the FeHex and MnHex distribution into the injured as well as in the healthy brain, we treated the animals after MCAO injury and measured the levels of the compounds in both the left (not injured) and right (injured by MCAO) brain hemispheres. As obtained in 3-day and 7-day PK studies (Figure 4 and Figure 5), FeHex levels are higher in plasma and lower in the liver than those of MnHex (Figure 6).

Importantly, similar brain levels of FeHex as of MnHex were found in the left uninjured brain, and those are similar to the levels determined in brain in 3-day and 7-day PK studies (Figure 4; Figure 5) when drugs were given via the SC route to healthy animals. Further higher levels of FeHex than of MnHex were found in the injured right hemisphere. The levels of both compounds were higher in the injured right than in the left uninjured hemisphere, presumably due to the damaged tissue lacking a functional blood–brain barrier. Such data would imply that both compounds should provide protection during an MCAO ischemia/reperfusion model of oxidative stress. Yet, as shown below, the efficacy studies told us otherwise.

### 4.2. Efficacy Studies

We conducted two MCAO studies. The first, a dose-dependent 3-day outcome study in a mouse MCAO model, aimed to define the dose to be used subsequently in a second study, a 7-day rat MCAO model.

#### 4.2.1. The 3-Day Mouse MCAO Study

**Figure 7:**
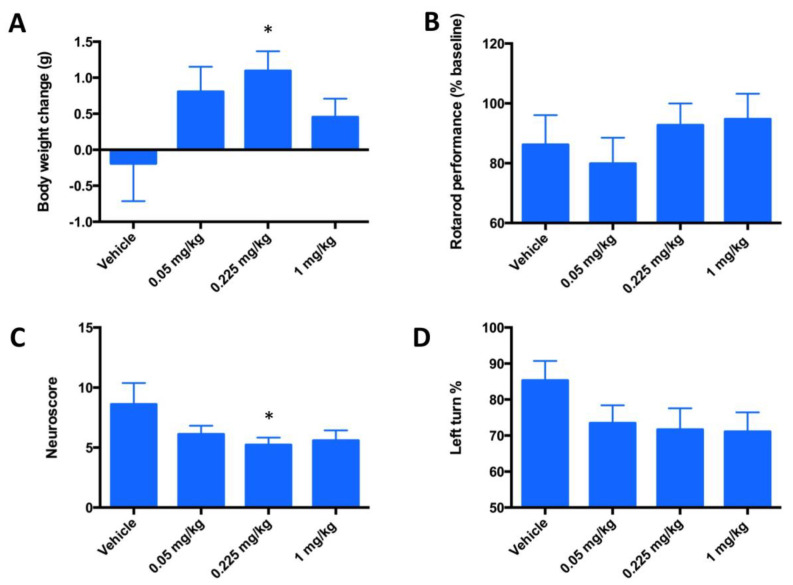
Evaluation of the neurologic deficit in a 3-day mouse MCAO stroke model in terms of motor neuron activities. Mice (*n* = 20) underwent a 90-min occlusion. FeHex was given IV via the tail vein at three different doses starting at 90 min post ischemia/reperfusion and continued twice daily for 3 days. (**A**) Body weights are expressed as body weight at 3 days post-stroke - body weight at surgery day; (**B**) rotarod performance, data are presented in % and are calculated as [(reading after injury)/(reading before surgery-baseline reading)] × 100; (**C**) neuroscore; and (**D**) Corner test. Vehicle vs. 0.225 mg/kg, *p* = 0.0347 for body weight change (**A**); *p* = 0.59 for rotarod (**B**); *p* = 0.047 for neuroscore (**C**); and *p* = 0.09 for Corner test (0.225 mg/kg), and *p* = 0.0725 for Corner test (1 mg/kg) (**D**). For all other concentrations vs. vehicle, *p* > 0.05; i.e., no significance was achieved. The “star” (*) symbol denotes statistically significant difference relative to the vehicle group (*p* < 0.05).

The design of a 3-day MCAO study was done at 3 doses out of which a dose of 0.225 mg/kg was the one used in a 3-day PK study. This dosing showed similar levels of FeHex and MnHex in the brain. We therefore anticipated similar therapeutic effects of both compounds. The 3-day mouse MCAO study was marginally enthusiastic, suggesting that FeHex may be efficacious in suppressing stroke injury. Drug injections were given IV via the tail vein and started at 90 min post ischemia/reperfusion and continued for 3 days twice daily at 0.05, 0.225, and 1 mg/kg. Data on rotarod test, body weights, corner test, and neuroscore are shown in Figure 7. FeHex improved the body weights of rats and neuroscore at 0.225 mg/kg, while a trend towards significance (*p* = 0.075) was seen with the corner test at 1 mg/kg.

**Figure 8:**
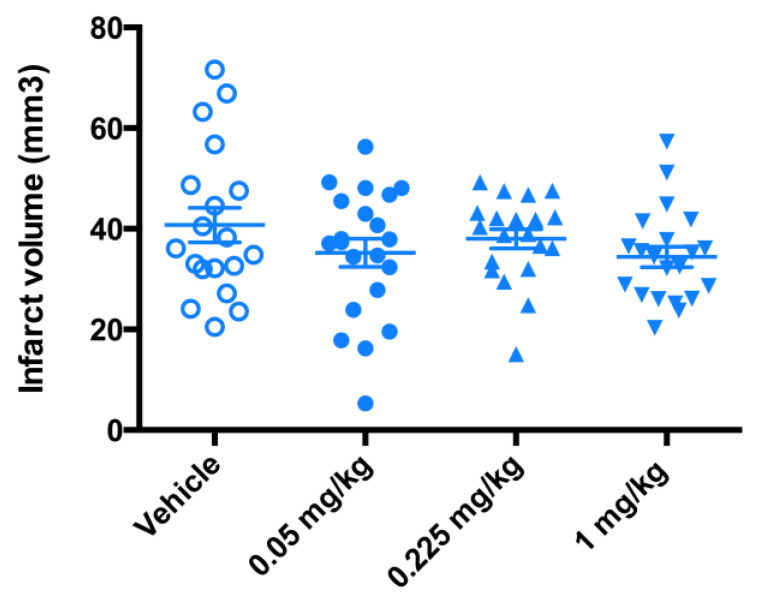
Evaluation of the infarct volume in a 3-day mouse MCAO stroke model. Mice (*n* = 20) underwent a 90-min occlusion. FeHex was given IV via the tail vein at three different doses starting at 90 min post reperfusion and continued twice daily for 3 days as indicated in the plot. No significant change in the infarct volume relative to the vehicle was found with either concentration. FeHex 0.225 mg/kg vs. Vehicle, *p* = 0.48.

While the trend towards protective effect was observed (Figure 7), the significant therapeutic benefit in terms of the reduction in infarct volume size was not demonstrated (Figure 8). The data from a 3-day mouse MCAO suggested a dose of 0.225 mg/kg to be used in a 7-day rat MCAO efficacy study.

##### 4.2.2. The 7-Day Rat MCAO Study

**Figure 9:**
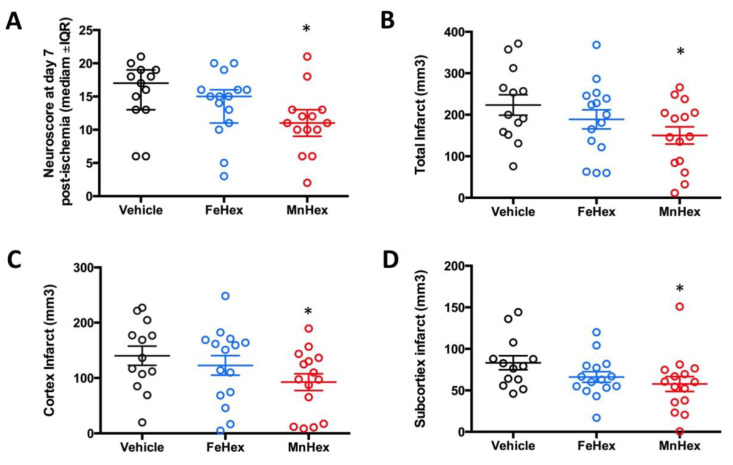
Evaluation of the neurologic outcome in terms of neuroscore and infarct volumes in a 7-day rat MCAO stroke study. Rats were either non treated (vehicle) or treated with FeHex and MnHex at 0.225 mg/kg twice per day for 7 days (*n* = 15–16). The first dose was given slowly IV via the tail artery at 5 min after reperfusion and the subsequent doses were given SC. At day 7 post-injury, a person blind to the treatment groups assessed the neurological deficit, at which point brains were harvested for infarct volume measurement. Neuroscore (**A**): *p* = 0.016 MnHex vs. Vehicle; *p* = 0.28 FeHex vs. Vehicle. Total infarct volume (**B**): *p* = 0.029 MnHex vs. Vehicle; *p* = 0.31 FeHex vs. Vehicle. Cortex infarct volume (**C**): *p* = 0.046 MnHex vs. Vehicle; *p* = 0.48 FeHex vs. Vehicle. Subcortex infarct volume (**D**): *p* = 0.049 MnHex vs. Vehicle; *p* = 0.11 FeHex vs. Vehicle. The “star” (*) symbol denotes statistically significant difference relative to the vehicle group (*p* < 0.05).

The design of the rat 7-day rat efficacy study closely resembles the rat 7-day PK study, where similar levels of FeHex and MnHex were achieved in the brain (Figure 6). In a 2-h rat PK study, under conditions of MCAO, FeHex was at a higher level in the injured right hemisphere than MnHex, while the levels of both compounds in the uninjured hemisphere were similar. Thus, FeHex should afford protection against brain ischemia/reperfusion injury. While FeHex treatment only slightly, but insignificantly, improved the neurological deficit, MnHex significantly improved the neurological deficit based on both the neuroscore and infarct volume measurements (Figure 9).

## 5. Discussion

Mechanistic studies of Fe porphyrins as SOD mimics [4,22], and peroxynitrite scavengers [22,23], and studies on their therapeutic efficacies were reported [24,25,26,27,28,29,30]. However, the pharmacokinetics on Fe porphyrins has not thus far been reported, though phase I clinical trials have been announced for an Fe porphyrin INO-4885 (WW-85) (that bears *ortho* pyridyl benzoate substituents, -CH_2_-C_6_H_4_COO^−^) for contrast-induced nephropathy [31].

We report here, for the first time, the pharmacokinetics (PK) of an Fe porphyrin, FeHex, which we compared to the PK of the analogous Mn porphyrin, MnHex. We further compared these two metalloporphyrins on the efficacy in a rodent stroke model. Regardless of its superb redox properties in an aqueous system and lipophilicity, favorable plasma PK, and clearly established distribution into the brain, we were unable to demonstrate the therapeutic efficacy of FeHex in an MCAO stroke model. MnHex, however, at the same brain levels as in the case of FeHex (despite much less favorable plasma pharmacokinetics), clearly demonstrated significant efficacy in the same stroke model.

In 3-day and 7-day PK studies in healthy rats, the levels of MnHex and FeHex were similar and in a range of 17 to 26 nM. In a 2-h PK/efficacy study where rats underwent MCAO injury (porphyrins were given via the mixed IV/SC route), the brain tissue contained significantly higher levels of FeHex than MnHex in the injured (right) brain hemisphere (~55 nM vs. ~35 nM, respectively); the levels of both compounds were similar in the uninjured brain hemisphere Figure 6). Yet, only MnHex, and not FeHex, was efficacious in a MCAO stroke model. Given the in vitro identical SOD activities of FeHex and MnHex, there must be a reason (other than their identical redox properties, *E*_1/2_ and log *k*_cat_(O_2_^●−^)) to account for the inefficacy of FeHex. We contemplate here that the inefficacy may be due to the in vivo interaction of FeHex (but not MnHex) with other biomolecules as a result of the greatly different coordination chemistry and biology of Fe vs. the Mn porphyrin center. Namely, in an aqueous solution, the Mn center has only weakly coordinated water. Conversely, the Fe center very much favors the axial coordination, and in aqueous solution, it binds strongly the hydroxo ligand in an axial position. We reported that in Fe(III) *N*-alkylpyridylporphyrins, hydroxo groups can be replaced with imidazolyl, while another imidazolyl molecule sits in a trans position [4]. Thus, it is probable that in a complex biological matrix, such as the brain, the Fe center may bind biological molecules, which would preclude its redox activity. Indeed, we found no significant SOD-like activity of FeTM-2-PyP(1-MeIm)2 and FeTM-4-PyP(1-MeIm)2 despite 220 mV more positive *E*_*1*/*2*_ for the Fe^III^/Fe^II^ redox couple of the bis(imidazole) complexes than their monohydroxo analogues (MeIm stands for methylimidazole) [4]. (OH)FeTM-4-PyP^4+^ is a *para* (indicated by number 4) isomer of (OH)FeTM-2-PyP^4+^, which is a methyl analog of FeHex. The imidazole groups are present in important biological building blocks, such as histidine and the related hormone histamine. Histidine is present in many proteins and enzymes and plays a vital part in the structure and binding functions of hemoglobin [32]. Further, imidazole 4-acetic acid (IMA) is a naturally occurring metabolite in the brain and oxidation product of histamine. IMA has pronounced neuropharmacological properties, many of which are consistent with the activation of γ aminobutyric acid receptor (GABA_A_) [33]. Imidazole also reportedly has an excitatory action on the evoked transmitter release from the phrenic nerve and on potassium-stimulated ^45^Ca uptake by synaptosomes in the rat [34].

The estimated half-life of FeHex (~23 h) is markedly higher than in the case of MnHex (~1.4 h). The above discussed propensity of FeHex, but not MnHex, to axial binding may also be responsible for its binding to plasma proteins, such as albumin histidine, cysteine and lysine sites [35,36,37,38], purine and pyrimidine nucleotides [39], and nucleic acids [40], which would prolong its plasma half-life. FeHex is an *ortho* isomeric alkylpyridylporphyrin with four positional atropoisomers [17]. Since long hexyl chains may preclude an interaction with proteins, one may expect that the interacting species of FeHex would be predominantly αααα or αααβ isomers with all four or three chains above or below the porphyrin plane.

The low level of FeHex in the liver may be due to: (i) its binding to the plasma proteins, which prevents the non-specific interaction of a highly charged molecule with liver components, and (ii) the higher propensity of FeHex than MnHex to undergo oxidative degradation by H_2_O_2_ produced in the liver [4,13]. Our PK results (long plasma half-life) suggest protein binding to be the main mechanism to account for the low liver FeHex levels. This is an important finding, which should be further investigated in preventing liver, kidney, and spleen retention of metalloporphyrin drug candidates.

The results from this study and from our earlier studies [4,13] indicate that the development of Fe porphyrins towards clinical needs thorough reconsideration due to the complex redox and coordination chemistry/biology, oxidative degradation, and possible Fenton-based toxicity. Thus, we are continuing with the exploration of the clinical potential of Mn porphyrins for ischemic stroke therapy. Two analogs, (H_2_O)MnTE-2-PyP^5+^ (BMX-010) and (H_2_O)MnTnBuOE-2-PyP^5+^ (BMX-001), are now in several phase II clinical trials [1].
